# Improving panicle blast resistance and fragrance in a high-quality *japonica* rice variety through breeding

**DOI:** 10.3389/fpls.2024.1507827

**Published:** 2025-01-13

**Authors:** Junhua Ye, Kai Wang, Yi Wang, Zhipeng Zhao, Ying Yan, Hang Yang, Lixia Zhang, Zejun Hu, Zhenying Shi, Dapeng Sun, Jianjiang Bai, Liming Cao, Shujun Wu

**Affiliations:** ^1^ Key Laboratory of Germplasm Innovation and Genetic Improvement of Grain and Oil Crops (Co-construction by Ministry and Province), Ministry of Agriculture and Rural Affairs, Crop Breeding and Cultivation Research Institute, Shanghai Academy of Agricultural Sciences, Shanghai, China; ^2^ Shanghai Agricultural Products Preservation Processing Engineering Technology Research Center, Shanghai, China; ^3^ Zhongken Seed Industry Co., Ltd., Shanghai, China; ^4^ Shanghai Agricultural Science and Technology Service Center, Shanghai, China

**Keywords:** pyramiding, panicle blast resistance, fragrance, eating and cooking quality, re-sequencing, rice

## Abstract

**Introduction:**

Huruan1212 (HR1212) is well-regarded for its superior eating and cooking quality in the lower reaches of the Yangtze River in China. Still, its high susceptibility to rice panicle blast and lack of fragrance have limited its further spread and utilization. *Pigm* and *Pi-ta* are two dominant genes known for their stable broad-spectrum resistance against rice blast fungus *Magnaporthe oryzae*, while *badh2* is the crucial gene that regulates rice aroma.

**Methods:**

In this study, we utilized a molecular marker-assisted selection backcrossing strategy to introduce *Pigm*, *Pi-ta*, and *badh2* into introgressed lines employing re-sequencing for precise genetic background selection.

**Results:**

Finally, we selected three introgressed lines, including two that carry *Pigm* with the highest background recovery rates, showing eating and cooking qualities similar to those of HR1212, and one line that pyramids *Pigm*, *Pi-ta*, and *badh2*, which features a strong aroma. They all displayed significantly enhanced resistance to panicle blast and improved yield compared to HR1212.

**Discussion:**

In conclusion, this study expanded the germplasm resources of *japonica*, providing a material foundation for enhancing breeding programs aimed at developing rice blast-resistant and high-quality fragrant *japonica* varieties. Additionally, the study demonstrated that integrating molecular markers and re-sequencing can inform breeders’ decision-making more precisely and efficiently.

## 1 Introduction

Rice is the staple food for over half of the world’s population and is one of the major food crops in China (Wing et al., 2018). According to 2022 statistics, its cultivation area accounts for a quarter of the annual planting area of grain crops in China (http://www.stats.gov.cn/). The development of new rice varieties is a key driver of rice production. To date, breeding high-yield, fine-quality, and disease-resistant varieties have remained rice breeders’ primary objective (Ye et al., 2022).

Rice blast disease caused by *Magnaporthe oryzae* (*M. oryzae*) is one of the most devastating worldwide. It is estimated that rice blast accounts for an annual loss of about 10%-30% in global rice yield, posing a significant threat to stable rice production (Hwang et al., 1987; Talbot, 2003). Rice blast manifests with different symptoms according to the affected organ, including leaf blast and panicle blast, the latter being particularly detrimental to yield (Gao et al., 2023). Recent studies have elucidated the genetic basis of rice blast resistance, and a total of 31 genes have been cloned, including two notable dominant genes, *Pigm* and *Pi-ta*, which confer durable, broad-spectrum resistance to panicle blast (Li et al., 2019, 2020; Song et al., 2024; Xiao et al., 2020). *Pigm*, located on chromosome 6, is either tightly linked or allelic to resistance gene *Pi2* and *Pi9* (Deng et al., 2006). Notably, *Pigm* has been shown to exhibit long-lasting resistance to *M. oryzae* without yield penalty (Deng et al., 2017; Wu et al., 2017; Zhai et al., 2022). A functional marker, M143104, was developed for *Pigm* and applied to breeding programs (Li, 2022; Wang et al., 2023).

*Pi-ta* is a single-copy gene near the centromere of chromosome 12, and the SNP at 2752 nt is the key to distinguishing the resistant *Pi-ta* allele from the susceptible *pi-ta* allele (Bryan et al., 2000). Based on this, several dominant markers were developed for genotyping *Pi-ta* (Huang et al., 2023; Jia et al., 2002, 2004; Wang et al., 2007), among which molecular markers YL155/YL87 and YL183/YL87 have been widely used in blast resistant germplasm screening (Khan et al., 2018; Liang et al., 2017; Ma et al., 2023; Terensan et al., 2021). Primers YL155 and YL183 were located in the intron region of the gene with the difference in their last four nucleotides, with YL183 specific for the susceptible allele, while YL155 specific for the resistant allele. However, the application of *Pi-ta* and *Pigm* in rice varieties remains limited. A study revealed that only 33%-55% of landraces or bred varieties in China carry the resistant genotypes of *Pi-ta* (Xu et al., 2020), and *Pigm* was not present in *japonica* varieties released in Jiangsu Province until 2020 (Wang et al., 2020), which highlighted the necessity of introducing the resistant allele(s) into more commercial rice varieties through breeding programs.

Aroma (fragrance) is a crucial component of end-use quality in rice and is largely controlled by allelic variation at the *badh2* gene (Bradbury et al., 2005). A mutation in the *BADH2* gene, which encodes betaine aldehyde dehydrogenase, leads to a loss of enzyme activity, resulting in the accumulation of 2-acetyl-1-pyrroline (2-AP). 2-AP is a potent flavor compound responsible for the distinctive aromas of both basmati and jasmine rice (Buttery et al., 1982). To date, 15 functional haplotypes have been identified within the coding region of *BADH2*, each with distinct geographical distribution patterns. The *badh2-E2.1* haplotype is predominantly found in *japonica* rice varieties in Jiangsu Province, with the 2-AP concentration in these varieties reaching approximately 660 ng/g (Pan et al., 2021). A specific molecular marker, InDel-E2, was developed to identify the *badh2-E2.1* haplotype, based on a 7-bp deletion in exon 2 of *badh2* (Wang et al., 2008).

Huruan 1212 (HR1212) is a *japonica* variety known for its exceptional taste quality, developed by the Crop Research Institute of the Shanghai Academy of Agricultural Sciences in 2017. It was awarded the Gold Medal in the first session of the Taste Quality Evaluation of High-Quality *Japonica* Varieties in China in 2018. Its outstanding eating and cooking quality (ECQ) has contributed to its popularity in Shanghai.

However, severe panicle blast symptoms have been observed in HR1212 in the breeding practices of recent years, which resulted in substantial yield losses. We analyzed the whole-genome variation of HR1212 and found that nine genes related to rice blast resistance (*Pid2*, *Pid3*, *Pi5-1*, *Pi-ta*, *RGA4*, *RGA5*, *Ptr*, *LHCB5*, *OsCERK1*) were all in susceptible genotypes, highlighting the necessity of improving blast resistance in HR1212 by introducing favorable alleles. Another drawback of HR1212 is its lack of desirable aroma, which may disadvantage it in the market among consumers seeking high-quality fragrant rice.

In this study, we aimed to enhance panicle blast resistance and aroma in HR1212 by developing introgressed lines through a molecular marker-assisted backcrossing strategy. Molecular markers were used for foreground selection, and re-sequencing was employed for background selection. The favorable alleles of *Pigm*, *Pi-ta*, and *badh2* were introgressed into HR1212, and superior introgressed lines were selected based on agronomic performance. As a result, three introgressed lines were successfully developed, exhibiting significantly improved panicle blast resistance, better yield performance, and high eating quality.

## 2 Materials and methods

### 2.1 Plant materials and field trials

HR1212 was the recurrent parent, lacking the favorable genotypes of *Pigm*, *Pi-ta*, and *badh2*. The donor parents included the *japonica* varieties Wuxianggeng 19 (WXG19) (carrying *Pi-ta* and *badh2*), Nangeng 46 (NG46) (carrying *badh2*), Songzaoxiang 1 (SZX1) (carrying *Pi-ta* and *badh2*), and a near-isogenic line (NIL) of Nangeng 9108 (hereafter referred to as the NIL) (carrying *Pigm*). NG46 has been widely used as a core parent in the lower Yangtze River region due to its excellent eating quality and high yield. SZX1 is a *japonica* variety known for its fragrant and glutinous texture. The NIL exhibits moderate resistance to rice blast disease. All parent lines were sourced from our genetic resources center.

Hybridization and backcrossing experiments were conducted from 2019 to 2022 in Fengxian District, Shanghai (30°54’N, 121°24’E), and Lingshui County, Hainan Province (18°30’N, 110°1’E). After backcrossing and marker-assisted foreground selection, selected lines were planted in Hainan and Shanghai in 2022 and 2023. The field trials followed local management practices, with row and column spacings set at 25 cm and 12 cm, respectively.

### 2.2 Molecular marker assays

Genomic DNA was extracted from fresh leaves of individual plants using the cetyltrimethylammonium bromide method. The genotypes of *Pigm* and *badh2* were identified using the markers M143104 (Wang et al., 2023) and InDel-E2 (Wang et al., 2008), respectively. The genotypes of *Pi-ta* were determined using two dominant molecular markers YL155/YL87 for *Pi-ta* and YL183/YL87 for *pi-ta* (Jia et al., 2002, 2004). The detailed information on these markers was listed in **Supplementary Table 1**. All of them were synthesized by Tsingke Biotech. Co. Ltd, Shanghai, China. The PCR assays were conducted in a total volume of 10 μL containing 1 μL of genomic DNA template (about 50 ng), 5 μL of 2×EasyTaq PCR SuperMix (TransGen Biotech Co. Ltd, Beijing, China), 0.5 μL of each primer (1 μmol/L), and 3 μL of ddH_2_O. PCR amplification was performed with the following profile: 94°C for 5 min, 35 cycles of 94°C for 45 s, primer annealing at different temperatures for 45 s (**Supplementary Table 1**), and 72°C for 90 s, and 72°C for 10 min. The PCR products of markers M143104, YL155/YL87, and YL183/YL87 were visualized by electrophoresis on 1%-2% agarose gels in 1×TAE buffer at 130 V for 15 to 30 min. The PCR products of InDel-E2 were separated in 8% non-denatured polyacrylamide gel (PAGE) in 1.0×TBE buffer followed by silver staining.

### 2.3 Whole-genome sequencing and variant identification

Sequencing libraries were constructed using 200 ng of DNA per sample. Paired-end 150 bp single-index sequencing was carried out using reagents from the MGISEQ-2000RS high-throughput sequencing instrument. FastQC (v.0.10.1) (https://www.bioinformatics.babraham.ac.uk/projects/fastqc/) was used to assess the quality of the short sequencing reads, and Trimmomatic (v.0.36) (Bolger et al., 2014) was employed to trim the reads. The Burrows-Wheeler Aligner software (BWA, v.0.7.8) (Li and Durbin, 2009) was used to align high-quality paired-end clean reads to the Nipponbare reference genome (MSU V7.0). The alignment results were processed using SAMtools (v.0.1.19) (Li et al., 2009). Variant detection was performed using GATK software (McKenna et al., 2010). Firstly, the default parameters were used for filtering. Then remove low-quality variants that meet the following criteria: QD < 20.0, QUAL < Mean QUAL, ReadPosRankSum < -8.0, FS > 10.0. Then variants are required to meet the following conditions: minor allele frequency (MAF) ≥ 4%, missing data rate < 20%.

### 2.4 Identification of genomic fragments in pedigree

The sliding window method was used to calculate the SNP similarity of genomic fragments between individuals and parents. The window size was set to 1,210 SNPs (with an average physical distance of 300 kb), and the step size was 121 SNPs. A sequence with more than 90% identical SNPs within a window was considered to originate from the parent. The distribution of putative genomic fragments was visualized using the *circlize* package in R (Gu et al., 2014).

### 2.5 Phylogenetic tree and genetic distance

The phylogenetic tree was constructed using FastTree software with the ‘JC+CAT’ model (Price et al., 2010). The identical-by-state (IBS) genetic distance between each pair of individuals was calculated using PLINK (v.1.9) (Purcell et al., 2007), and the results were visualized as a heat map using *pheatmap* package in R.

### 2.6 Evaluation of panicle blast resistance at the booting stage

At the early booting stage, the plants were injected with a mixed spore suspension of *M. oryzae* isolates, consisting of five representative strains (2023-35, 2023-197, 2023-207, 2023-261, and 2023-296) in equal proportions, with 30-40 spores per field of view under a 10 × 10 magnification microscope. The spore suspension was provided by the Institute of Plant Protection, Jiangsu Academy of Agricultural Sciences. Each plot was inoculated with 10 panicles, applying 1 mL of the suspension per panicle. The experiment was conducted at the Zhuanghang Experimental Station in Fengxian District, Shanghai. At maturity, the number of diseased grains and total grains per tagged panicle were recorded. The average percentage of symptomatic grains (PSG) was used to evaluate the severity of the panicle blast.

### 2.7 Identification of agronomic traits and yield-related traits

The phenotypic evaluation was conducted in Shanghai in 2023. Each line was planted in ten rows, with seven plants per row. The heading date was recorded when approximately half of the plants in each plot had headed. Plant height, number of grains per panicle, and seed-setting rate were measured according to the standard evaluation systems for rice (Yu and Wei, 2010) using five representative plants from the central plot. Yield per plant was calculated as the average yield of ten consecutive plants from the central plot.

### 2.8 Measurements of physicochemical properties related to ECQ

After harvest, the grain samples were stored for three months in a sample drying room. Approximately 100 g of rice grain samples were shelled using a seed hulling machine (JLG-II, Zhonggu Mechanical Instrument Co., Ltd., Zhengzhou, China) and then milled with a machine (JNM-III, China Grain Storage Chengdu Storage Research Institute Co., Ltd., Chengdu, China). The milled rice samples were kept in an environmentally controlled room at approximately 28°C (humidity <10%) for 7 days. Subsequently, part of the milled rice was ground into powder and screened through a 100-mesh sieve for further analysis.

Gel consistency and amylose content were determined according to departmental standard NY/T 83-2017 and national standard GB/T 15683-2008, respectively. The pasting properties of starch were assessed using a rapid viscosity analyzer (RVA4500, PerkinElmer Inc., United States) following regulations of the American Association of Cereal Chemists (Cereals & Grains Association, 2000) and the manufacturer’s instructions.

The concentration of 2-acetyl-1-pyrroline (2-AP) was measured using a GCMS-QP2020 NX gas chromatograph-mass spectrometer (Shimadzu, Japan), in accordance with departmental standard NY/T 4350-2023. The percentage of chalky grains (CGP) was determined using approximately 200 randomly selected whole-milled grains analyzed by the QM3 Grain Rice Analyzer (VIBE Imagine Analytics, Israel). CGP was calculated as follows: CGP (%) = (Number of grains with at least 20% whitish area/Total number of grains) × 100%.

### 2.9 Statistical analysis

The Chi-square test was performed using SAS 9.4 software. Analysis of variance was conducted with R software, and the least significant difference method was employed for multiple comparisons.

## 3 Results

### 3.1 Development of introgressed lines for *Pigm*, *Pi-ta*, and *badh2*

Three populations were developed with HR1212 as the recurrent parent (**Supplementary Figure 1**). Population 1 (P1) was developed through continuous backcrossing of HR1212 with a stable line derived from the cross between NG46 and WXG19. The segregating BC_1_F_1_ population was screened using markers of *Pi-ta* and *badh2*. Since HR1212 lacks the favorable alleles of *Pigm*, *Pi-ta*, and *badh2*, these desirable genotypes were initially introduced as heterozygotes during the backcrossing process. Individual plants carrying heterozygotes of *Pi-ta* or *badh2* and having agronomic performance similar to HR1212 were chosen for further backcrossing. Population 2 (P2) was derived from the backcross of HR1212 to SZX1 with a similar selection process (**Supplementary Figure 2**, **Supplementary Table 2**). Population 3 (P3) was formed by backcrossing HR1212 with NIL, and the offspring individuals were screened using the molecular marker for *Pigm* (**Supplementary Figure 2**). Individual plants carrying heterozygotes of *Pigm* and having agronomic performance similar to HR1212 were chosen for further backcrossing. As selection based on agronomic performance began in the early generations, genotype segregation did not conform to the expected theoretical ratios (**Supplementary Table 3**).

The BC_5_F_1_ of P3 continued to backcross with HR1212 to produce BC_6_F_1_. Simultaneously, BC_5_F_1_ of P3 was crossed with BC_5_F_1_ of P1 and BC_5_F_1_ of P2. The progeny from these crosses were tested using molecular markers (**Supplementary Table 4**). Based on agronomic performance, 41 individual plants (C01-C41) were selected for further analysis (**Supplementary Table 5**).

### 3.2 Comparison of the genetic background between the introgressed individuals and HR1212

**Figure 1:**
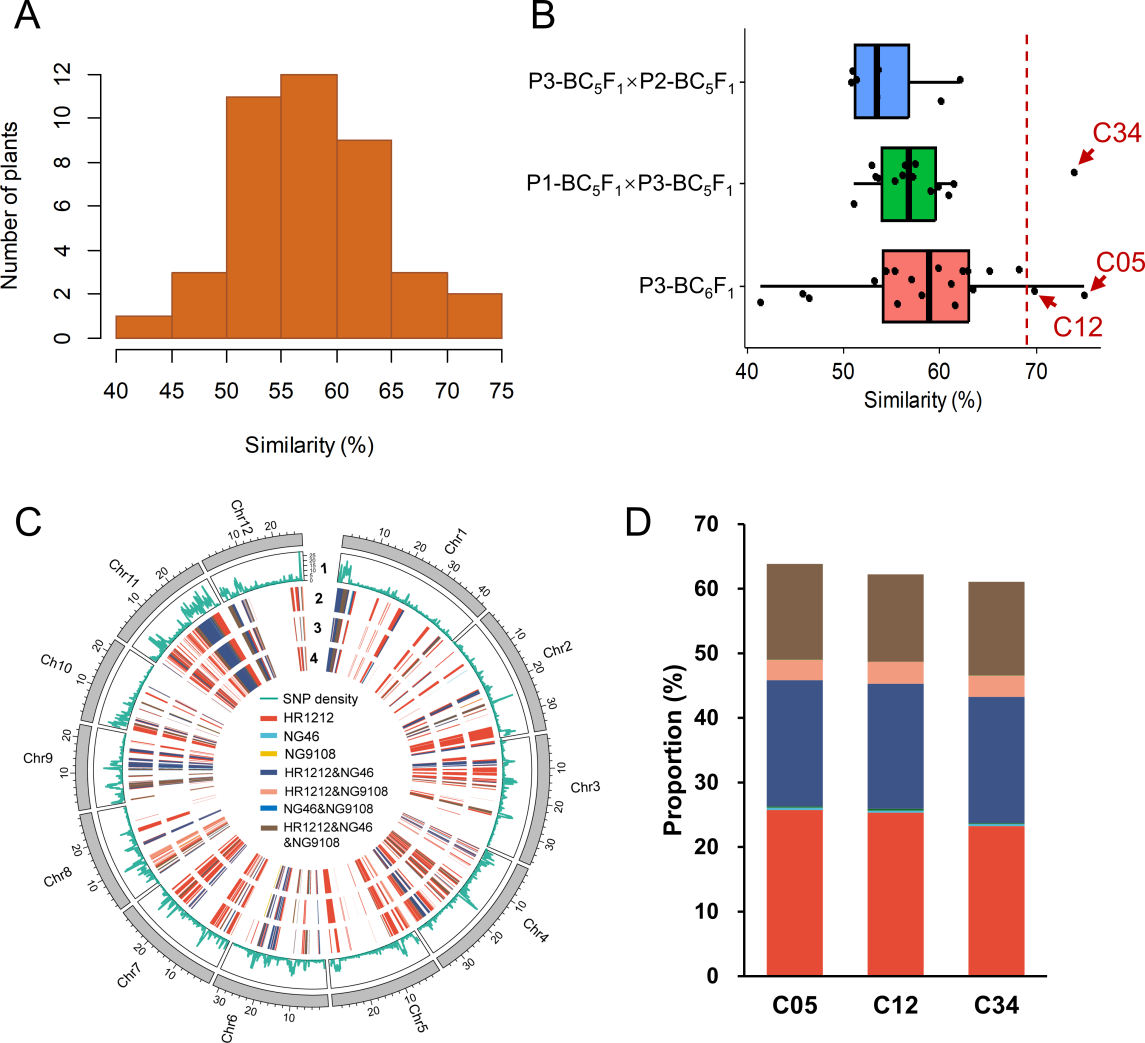
Analysis of genetic similarity between introgressed individuals and recurrent parent HR1212. **(A)** The distribution of SNP similarity between 41 individuals and recurrent parent. **(B)** Origin of individuals with various SNP similarities. The vertical red dotted line is located at 69% similarity. Three individuals with the highest similarity were C05 (74.91%), C34 (73.89%), and C12 (69.82%). **(C)** Distribution of genetic introgressed segments from parents on 12 chromosomes of three individuals. 1 indicates SNP density. From 2 to 4, the tracks represent individuals C34, C12, and C05, respectively. **(D)** The genomic contribution of parents to the three individuals. The same color scheme was used as **(C)**.

To investigate the recovery of the genomic background, we conducted whole-genome sequencing with an average depth of 13.86× for 41 introgressed individuals (C01-C41) and deep resequencing for the recurrent parent HR1212 (**Supplementary Table 6**). SNP similarities between the 41 introgressed individuals and HR1212, based on 1,130,432 SNPs distributed across 12 chromosomes, showed a normal distribution (**Figure 1A**; **Supplementary Figure 3**). Among the three crosses, BC6F1 of P3 (P3-BC_6_F_1_) exhibited the highest genetic background similarity with HR1212 (**Figure 1B**). Three individuals, including two (C05 and C12) from P3-BC_6_F_1_ and one (C34) from P1-BC_5_F_1_×P3-BC_5_F_1_, showed the highest degree of genetic background recovery (**Figure 1B**). Phylogenetic tree and genetic distance analyses also showed that these three individuals were genetically closest to HR1212 (**Supplementary Figure 4**).

To elucidate the genomic composition of these three individuals, we analyzed the whole-genome variation of their parents—NG46, NG9108, and HR1212. Despite their different pedigrees, the genomic compositions of the three individuals were quite similar (**Figure 1C**). About 61.8% of the genomic segments of the three individuals were derived from HR1212, of which HR1212-specific fragments accounted for about 24.8%, followed by shared genetic components from HR1212 and NG46, constituting about 19.5%. The common genetic framework from all three parents represented around 14.3% (**Figure 1D**).

### 3.3 Enhanced panicle blast resistance of introgressed lines

**Figure 2:**
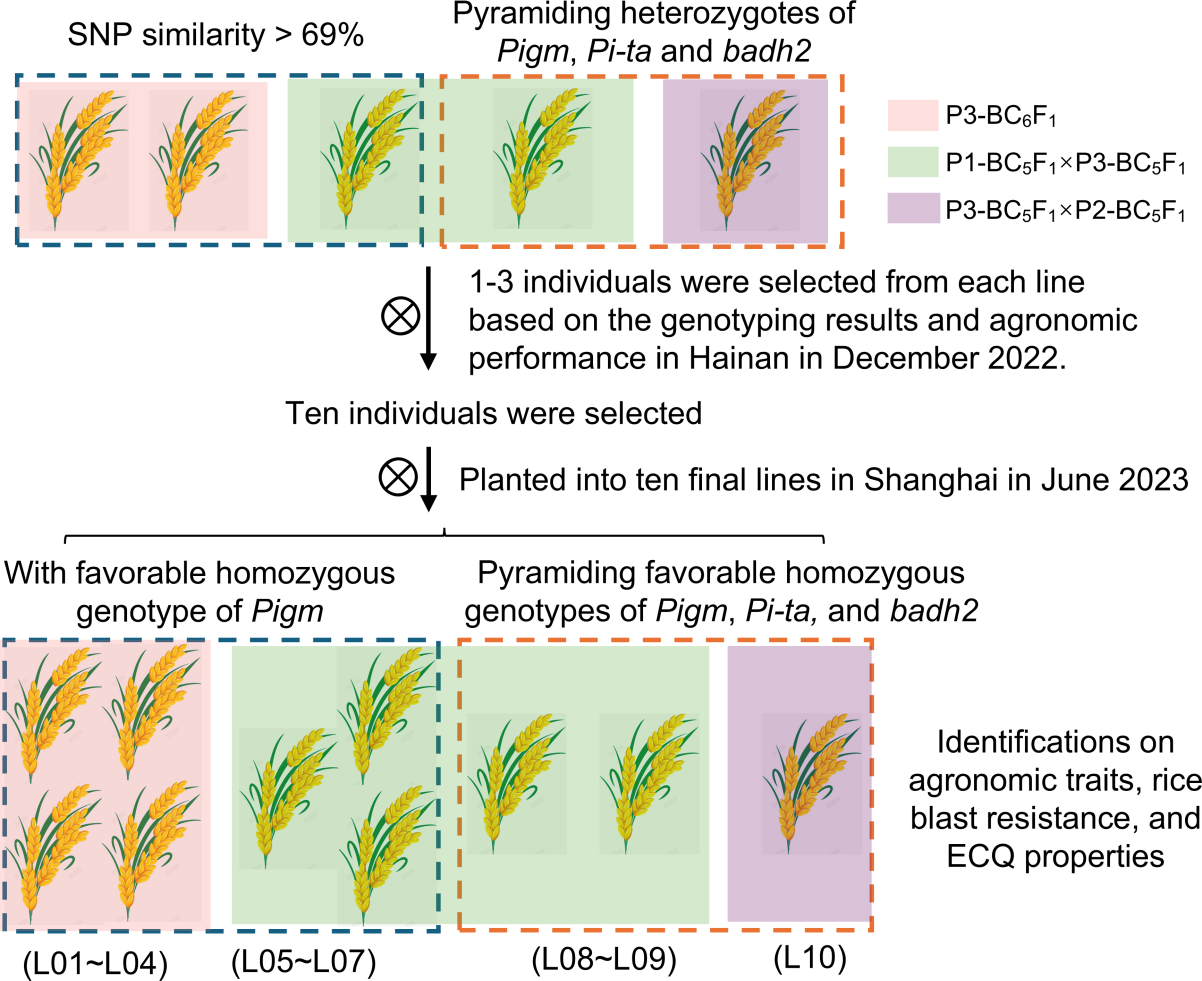
Flowchart of introgressed lines selection using two strategies. Three background colors of individuals or lines mean that they came from different hybrid combinations. The dashed boxes of different colors indicate the two strategies used for selecting the introgressed lines.

Two strategies were employed to select the introgressed lines: one focused on genetic background recovery following foreground selection, while the other prioritized the number of favorable alleles being pyramided. In December 2022, five targeted individuals were selected and planted into separate lines in Hainan (**Figure 2**). These included three individuals (C05, C12, and C34) with the closest genetic backgrounds to HR1212, as well as two individuals (C40 and C41) that pyramided heterozygotes of *Pigm*, *Pi-ta*, and *badh2* (**Figure 1B**). For the segregating population, further screening was conducted through genotyping and agronomic performance assessment in April 2023. Consequently, ten individuals were selected and planted into ten final lines in June in Shanghai. These comprised seven monogenic lines (L01-L07) with *Pigm* and three lines (L08-L10) that pyramided *Pigm*, *Pi-ta*, and *badh2* (**Figure 2**; **Supplementary Figure 5**).

**Figure 3:**
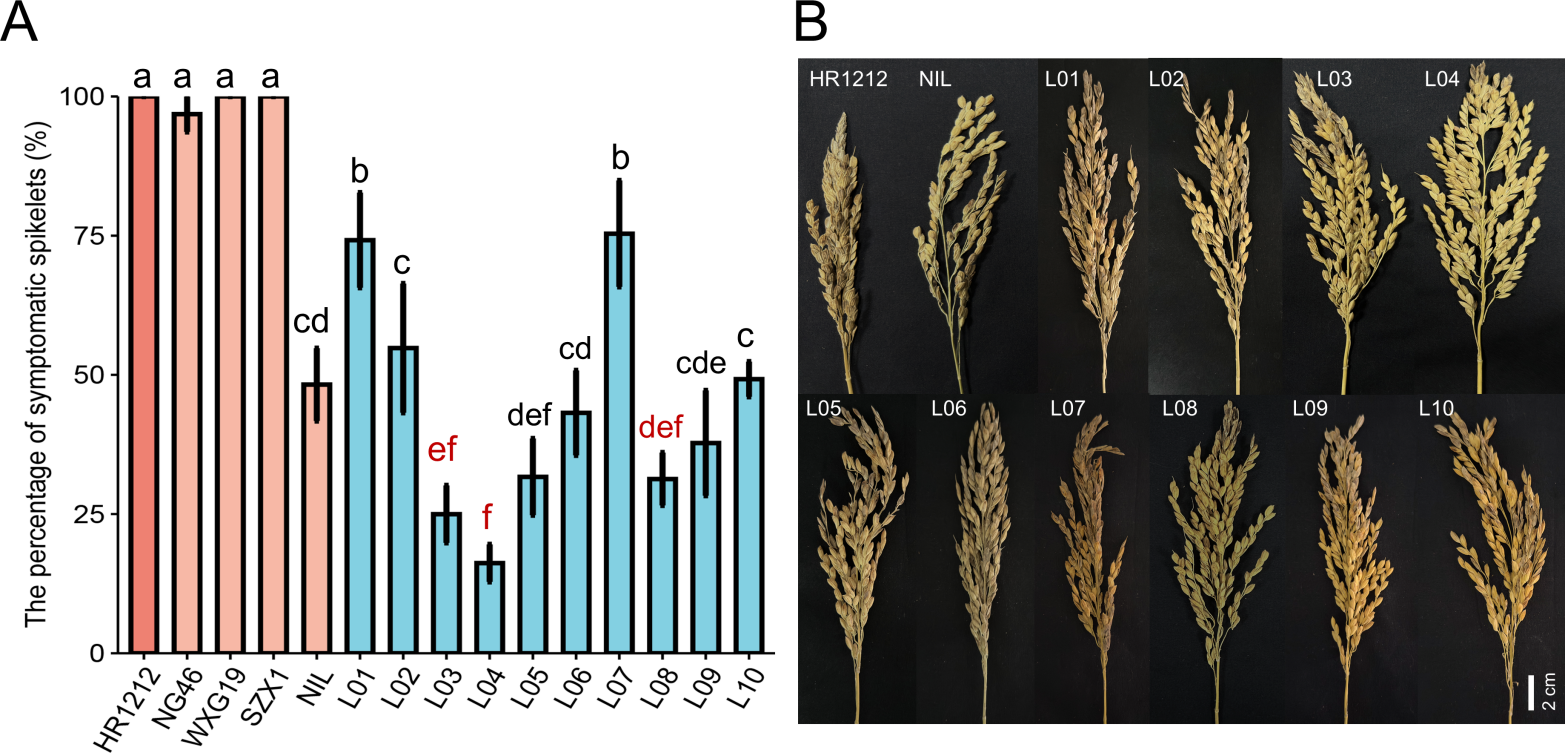
Panicle blast severity of the introgressed lines and the parents. **(A)** The incidence of introgressed lines (L01-L10) and five parents in the panicle blast evaluation. The red letters correspond to the three most resistant introgressed lines for panicle blast. Error bars, s.e. (*n*=10). Multiple comparison was conducted using the least significant difference (LSD) test. Different letters above the bars indicate significant differences among them (*P*<0.05). **(B)** Panicle blast symptom illustrations.

To evaluate the panicle blast resistance of the final introgressed lines, ten introgressed lines and five parent lines were inoculated with a mixture of *M. oryzae* isolates at the booting stage. The results indicated that all introgressed lines (L01-L10) displayed significantly improved panicle blast resistance compared to HR1212 (**Figure 3**). Notably, L03 and L04 exhibited significantly higher resistance than the donor parent NIL. Six lines (L02, L05, L06, L08, L09, L10) showed no significant differences in panicle blast resistance compared to NIL (**Figure 3**).

### 3.4 Comparison of agronomic traits between introgressed lines and HR1212

In Shanghai, heading dates for the ten introgressed lines ranged from 101 to 105 days. We focused on three lines (L03, L04, and L08) known for their strong resistance to panicle blast. Compared to the recurrent parent HR1212, both L04 and L08 exhibited a significant increase in plant height (**Supplementary Figure 6**). For grain yield-related traits, all three lines showed a significant increase in the number of secondary branches compared to HR1212, with L04 and L08 also demonstrating a notable increase in the number of grains per panicle. Additionally, the grain length-to-width ratio was higher in L03 and L08. The average yield per plant of the three introgressed lines exceeded that of HR1212 by more than 12% (**Supplementary Figure 7**).

### 3.5 Comparison of ECQ between introgressed lines and HR1212

**Figure 4:**
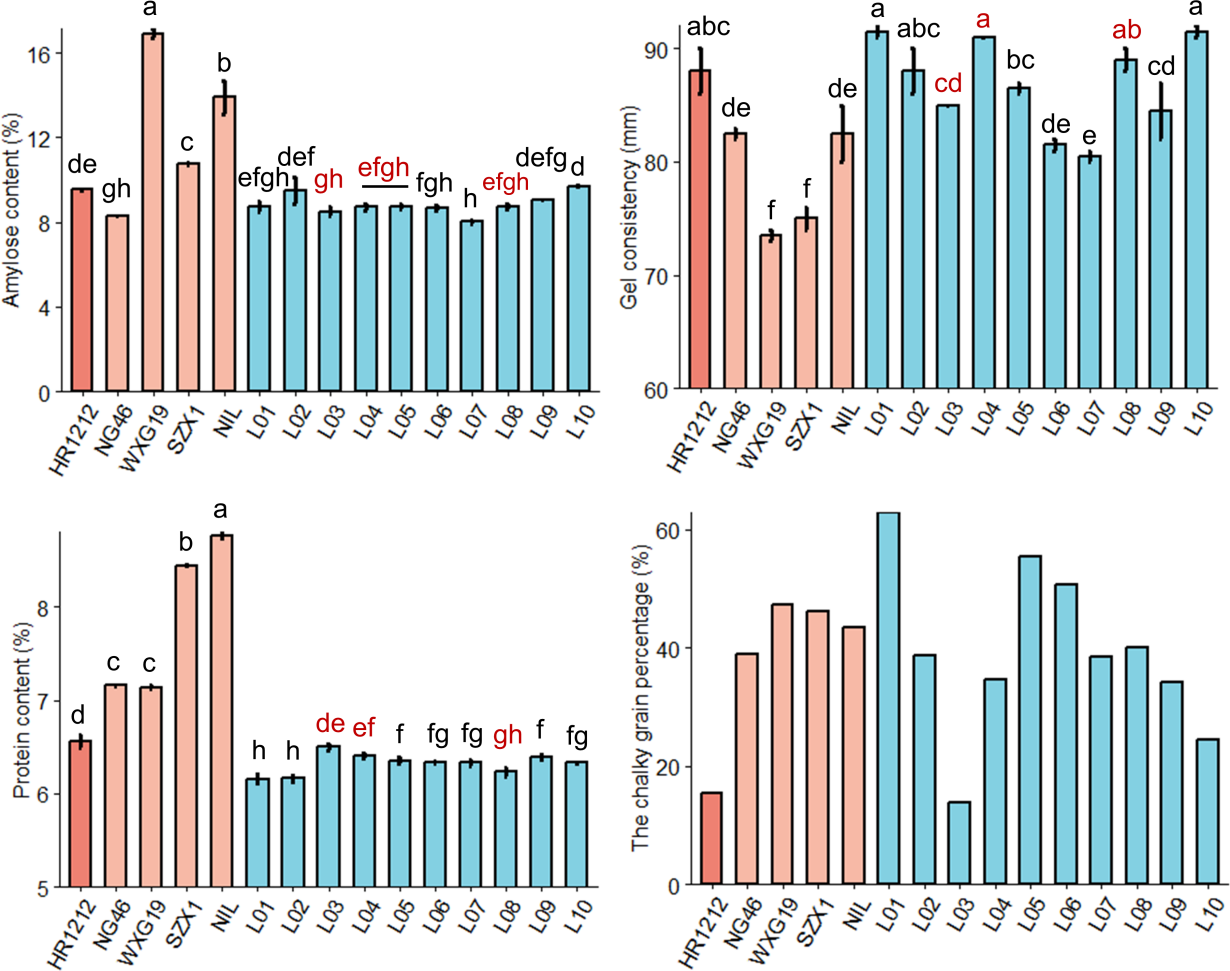
Quality traits evaluation of the introgressed lines and the parents. The red letters correspond to the three most resistant introgressed lines for rice blast. Error bars, s.e. (*n*=2). Multiple comparison was conducted using the least significant difference (LSD) test. Different letters above the bars indicate significant differences among them (*P*<0.05).

To evaluate the ECQ of the introgressed lines, several key physicochemical characteristics were assessed. Compared to HR1212, L03 exhibited significantly lower amylose content, gel consistency, and chalky grain percentage, while L04 and L08 showed significantly reduced protein content (**Figure 4**). The RVA profile indicated that the parameters for L03 and L04 were closer to HR1212 than those for L08 (**Supplementary Figure 8**). The CGP for L03 was 13.86%, slightly lower than HR1212’s 15.5%, while L04 and L08 had significantly higher CGP values (**Figure 4**).

Additionally, the 2-acetyl-1-pyrroline (2-AP) content in the milled rice revealed that the pyramiding line L08 had a 2-AP concentration of 711.83 ng/g, surpassing that of its fragrance gene donor, WXG19 (558.24 ng/g) (**Supplementary Figure 9**).

In conclusion, among the three introgressed lines exhibiting the highest resistance to panicle blast, the ECQ of L03 and L04 was more similar to that of HR1212 based on their physicochemical properties, with L03 outperforming HR1212 due to its lower CGP. L08 had lower protein content and similar amylose content compared to HR1212. Although its starch viscosity was inferior to that of L03 and L04, it possessed a strong fragrance (**Figure 4**; **Supplementary Figure 9**).

## 4 Discussion

High yield, good quality, and disease resistance are primary objectives in rice breeding. Recently, excellent eating quality has become a key determinant in the breeding of *japonica* varieties in China, driven by rising living standards. However, *japonica* varieties known for their superior eating quality have historically been susceptible to rice blast disease. Previous studies have identified genetic constraints, particularly the reallocation of genetic resources during hybridization and genetic linkage—especially those on chromosome 6 (a deleterious linkage between *Pigm*/*Piz-t* and *Wx^mp^*)—that impede the simultaneous achievement of high eating quality and rice blast resistance (Xiao et al., 2020, 2021). For example, we analyzed the whole-genome data and found that HR1212, a major *japonica* variety in Shanghai, has favorable alleles *Wx^mp^* and *ALK^b^* for eating quality, but also carries susceptible alleles of *Pigm*, *Pid2*, and *Pid3* for rice blast on chromosome 6. This highlights the necessity to overcome genetic linkage drag and introduce blast-resistant alleles.

During the backcrossing process, no negative drag was observed in this study, suggesting that unfavorable linkages, such as the *Pigm*-*Wx^mp^* linkage, might be broken through continuous positive selection, thereby providing a promising approach for refining elite varieties with minor drawbacks (Nihad et al., 2024; Tian et al., 2021). This strategy has been successfully applied to rice and other crops (Hu et al., 2023; Lv et al., 2014; Xiao et al., 2019). We observed that while all selected lines possessed the same superior alleles for *Wx* and *ALK* as HR1212, those selected based on genetic background recovery exhibited better eating quality compared to those selected solely for the number of pyramided alleles. This suggests that, in addition to the major genes *Wx* and *ALK*, HR1212’s genome might contain other genes influencing eating quality. Additionally, the RVA profile provides a more accurate assessment of eating quality than amylose content and protein content.

Previous studies have shown that the resistance conferred by pyramiding lines is not merely the sum of the resistance effects of individual genes (Fukuoka et al., 2015; Tabien et al., 2000). This is consistent with our findings, which indicate no significant difference in panicle blast resistance between the pyramiding lines of *Pigm* and *Pi-ta* and the *Pigm* monogenic lines. Several improved lines exhibited enhanced resistance to panicle blast compared to the donor NIL, possibly resulting from differences in genetic background between the improved lines and NIL (Feng et al., 2022). Some research suggests that lines with pyramided resistance alleles may experience reduced yield or other negative effects (Xiao et al., 2019). However, our study found no significant disadvantage in yield or eating quality for the pyramiding lines, likely due to the effective recovery of the genetic background achieved through backcrossing.

The reduced cost of sequencing has facilitated more efficient detection of genetic backgrounds using next-generation sequencing (NGS). Previous studies typically employed hundreds of SSR markers evenly distributed across the 12 chromosomes for background selection (Liu et al., 2016; Sabar et al., 2019; Wang et al., 2017; Xiao et al., 2019). In contrast, we used NGS to identify genome-wide variants and compared genetic backgrounds with greater accuracy and efficiency. This approach allowed us to precisely uncover the genomic contributions of both donor and recurrent parent genotypes. Additionally, our analysis revealed that the genomic components shared by HR1212 and NG46 accounted for approximately half of the genetic introgression, which aligns with the pedigree relationship of HR1212.

In this study, multiple backcrosses were conducted to ensure that the introgressed lines were as close as possible to the recurrent parent HR1212. However, a limitation is that the introgressed lines were only self-crossed twice. To develop exceptionally stable lines and further breed new varieties, additional self-crossing will be necessary.

In the genetic improvement, some major varieties may be replaced due to some shortcomings that fail to cater to contemporary breeders’ requirements. The present study offers valuable insight into enhancing the varieties of this sort. During backcrossing, those unfavorable traits were addressed using molecular markers, and a high recovery rate of the genetic background was ensured through NGS. This work demonstrates the optimization of breeding strategies, ultimately contributing to the improvement of a variety with high eating and cooking qualities. Furthermore, it points towards a promising future direction for breeding endeavors that are more precise, efficient, and sustainable.

## Data Availability

Raw genome re-sequencing reads are available under the National Center for Biotechnology Information sequence read archive with the BioProject ID: PRJNA1116583. The names of the repository/repositories and accession number(s) can be found in the article/**Supplementary Material**.
