# Supplementary material for: Improving panicle blast resistance and fragrance in a high-quality japonica rice variety through breeding
Source: Front Plant Sci. 2025 Jan 13;15:1507827. doi: 10.3389/fpls.2024.1507827 (PMC11770053; doi:10.3389/fpls.2024.1507827)
Supplement: Supplementary file 1 [file DataSheet1.docx]

Supplementary Material

# Supplementary Figures and Tables

## Supplementary Figures


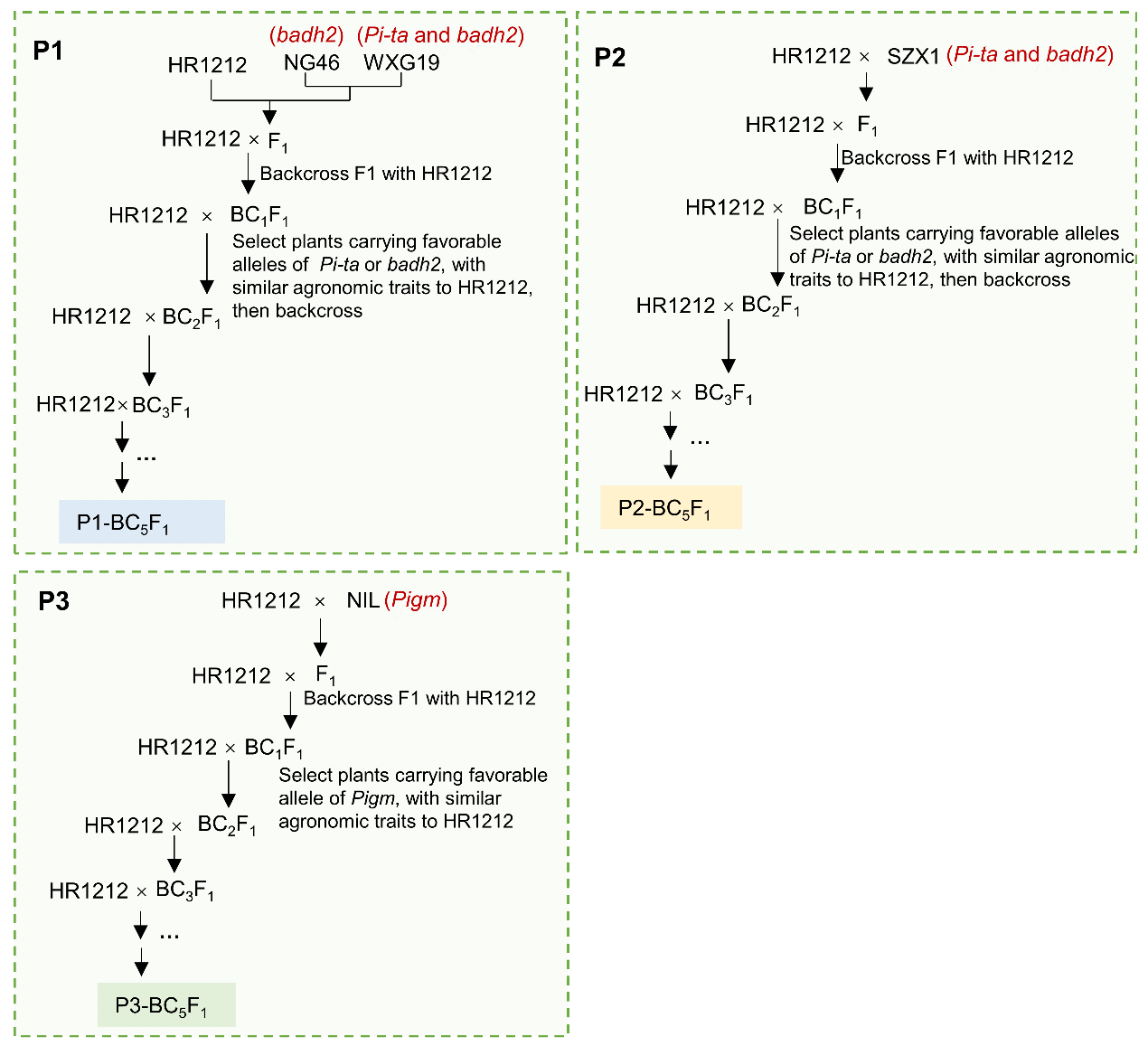


**Supplementary Figure 1.** Development scheme of three molecular marker-assisted backcross populations. P1, population 1; P2, population 2; P3, population 3. NIL here refers to a near-isogenic line of Nangeng 9108. The red letters highlight the superior alleles carried by each parent.


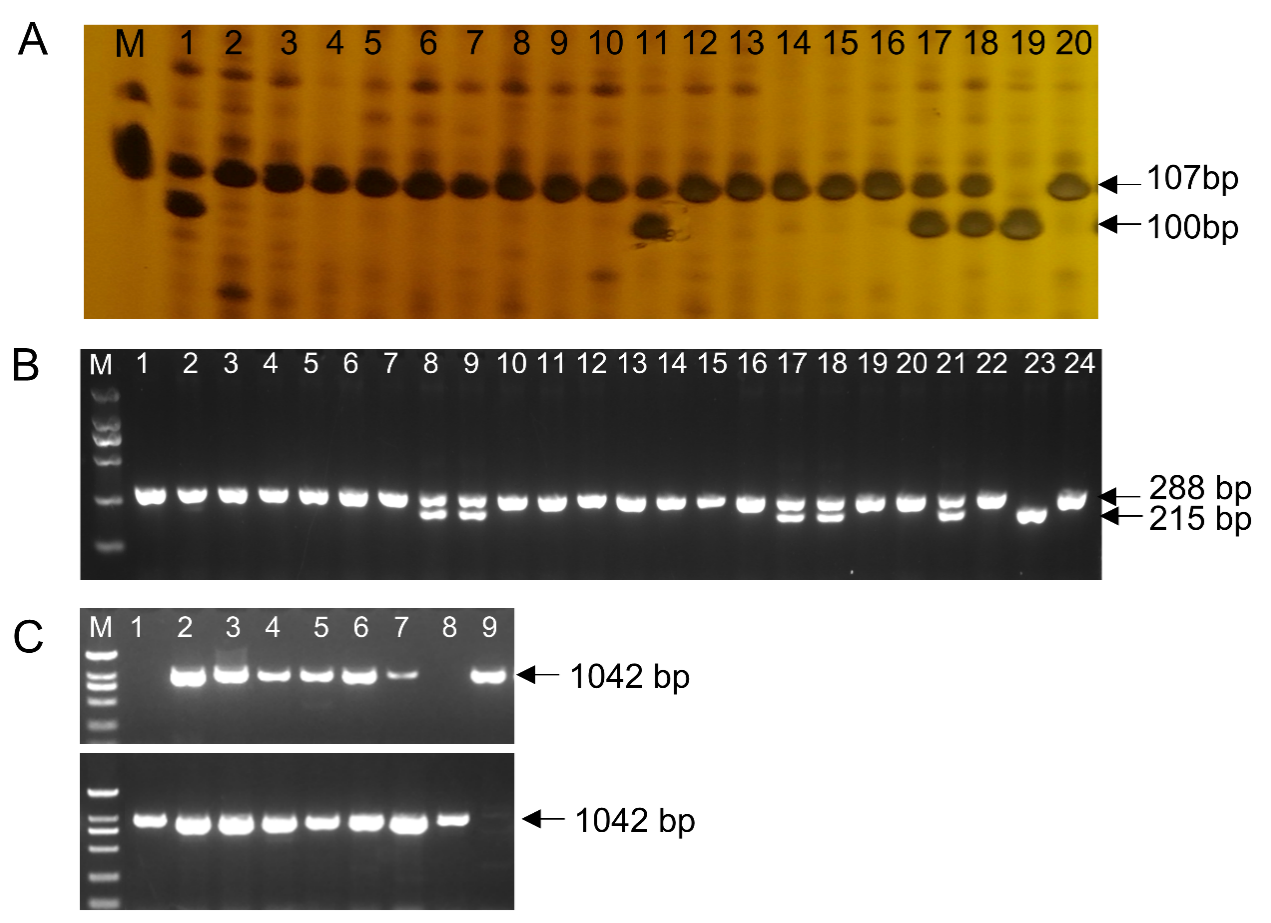


**Supplementary Figure 2.** Genotyping for *badh2*, *Pigm*, and *Pi-ta* in backcrossing populations using molecular markers. **(A)** The genotype of *badh2* in plants was identified by the molecular marker InDel-E2. M, marker DL2000; 1-18, plants from backcrossing population;19, donor parent WXG19 (fragrant); 20, HR1212 (non-fragrant). **(B)** The identification of *Pigm* using marker M143104. M, marker DL2000; 1-22, plants from backcrossing population; 23, donor parent NIL (resistant); 24, HR1212 (susceptible). **(C)** Primers YL155/YL87 of a resistant *Pi-ta* allele (top) and primers YL183/YL87 of a susceptible *pi-ta* allele (bottom) were used for polymerase chain reaction (PCR). M, marker DL2000; 1-7, plants from backcrossing population; 8, HR1212 (susceptible); 9, donor parent SZX1 (resistant).


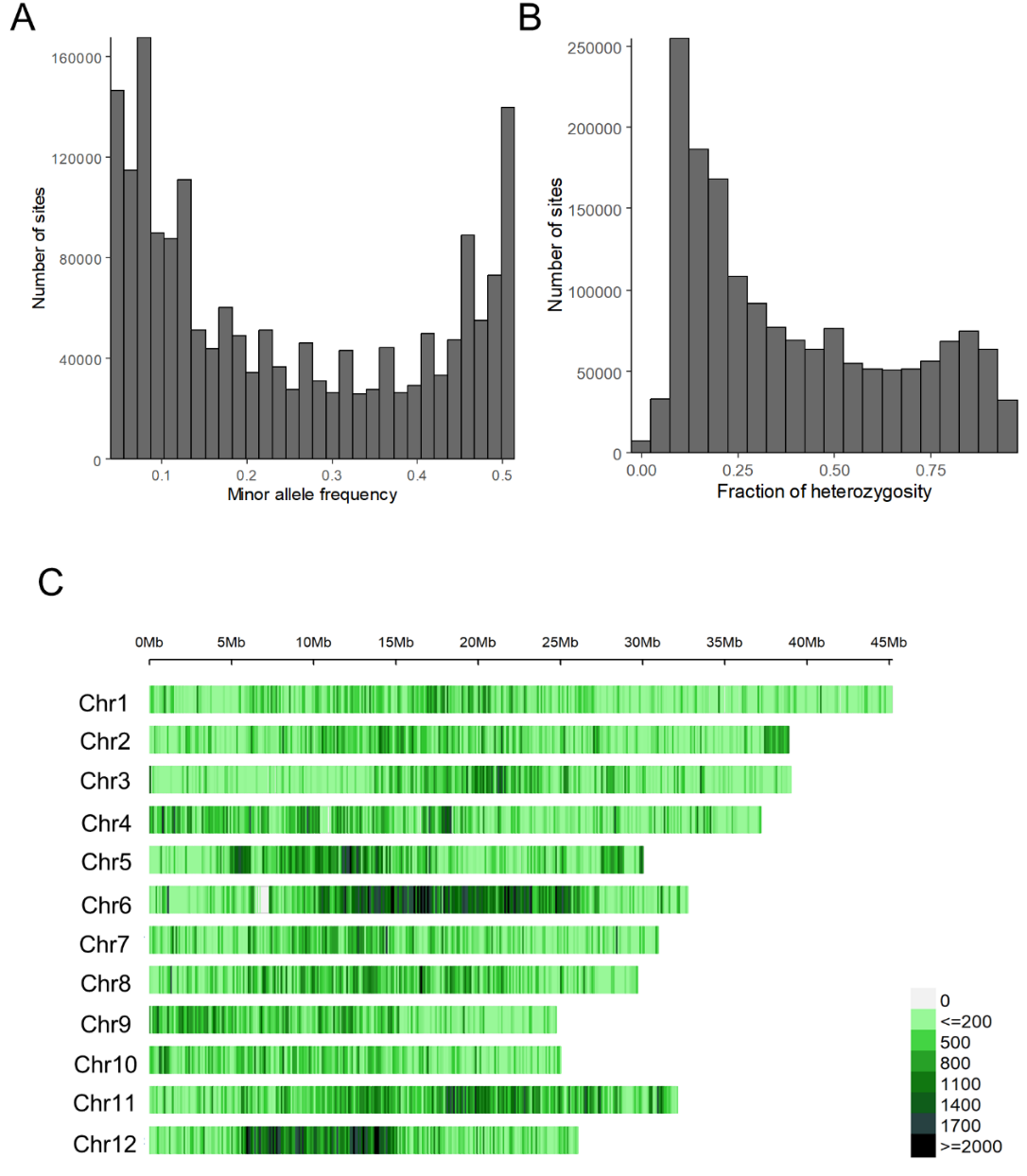


**Supplementary Figure 3.** Summary of resequencing data of 42 individuals. Distribution of minor allele frequency **(A)** and fraction of heterozygosity per site **(B)**. **(C)** Distribution of SNP density per 100 kb across the 12 chromosomes.


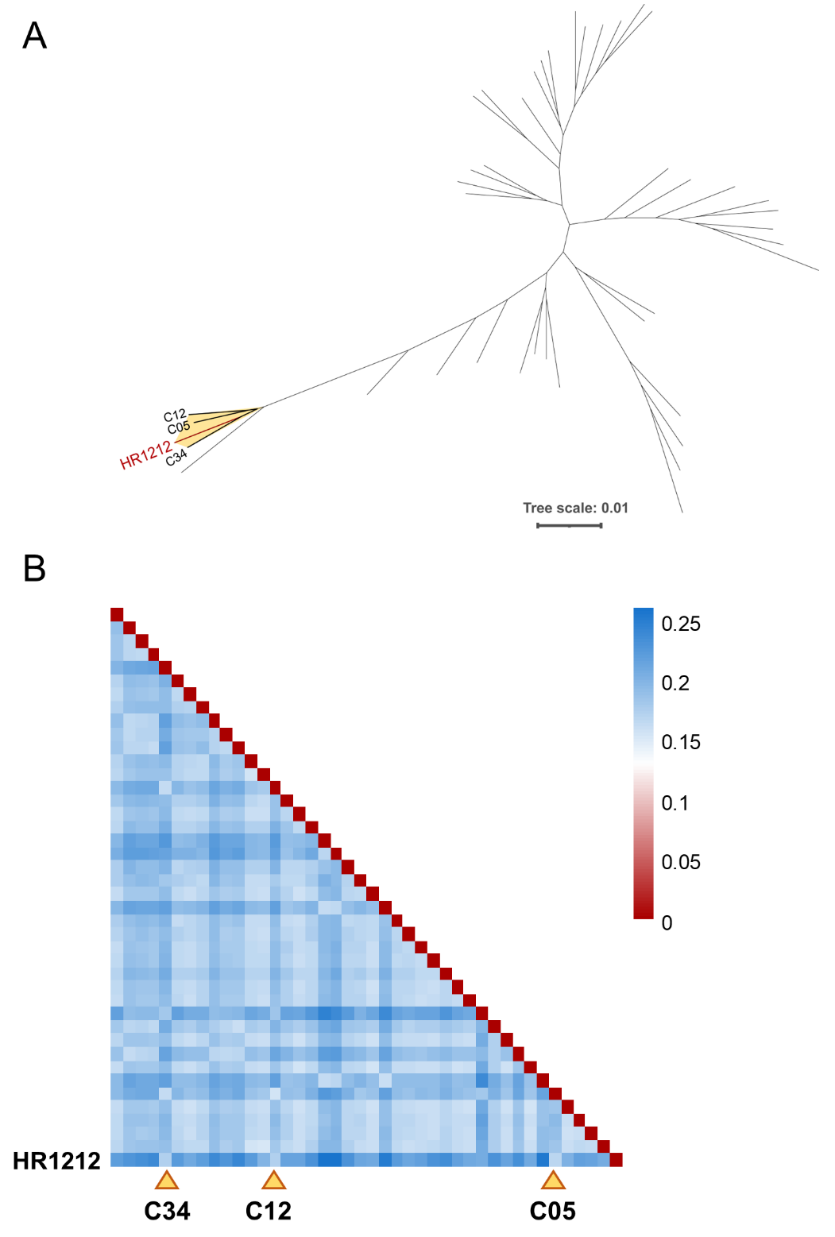


**Supplementary Figure 4.** Phylogenetic relationships and nucleotide distance among 41 individuals and their recurrent parent HR1212. **(A)** The approximately maximum-likelihood phylogenetic tree was built through the alignment of the nucleotide sequence. **(B)** Genetic distance was measured through identity by state (IBS) distance between each pairwise of individuals. The bottom row showed the genetic distance between HR1212 and 41 individuals, with the three individuals pointed to by the triangular markers being the lightest in color, which are closest in genetic distance to HR1212.


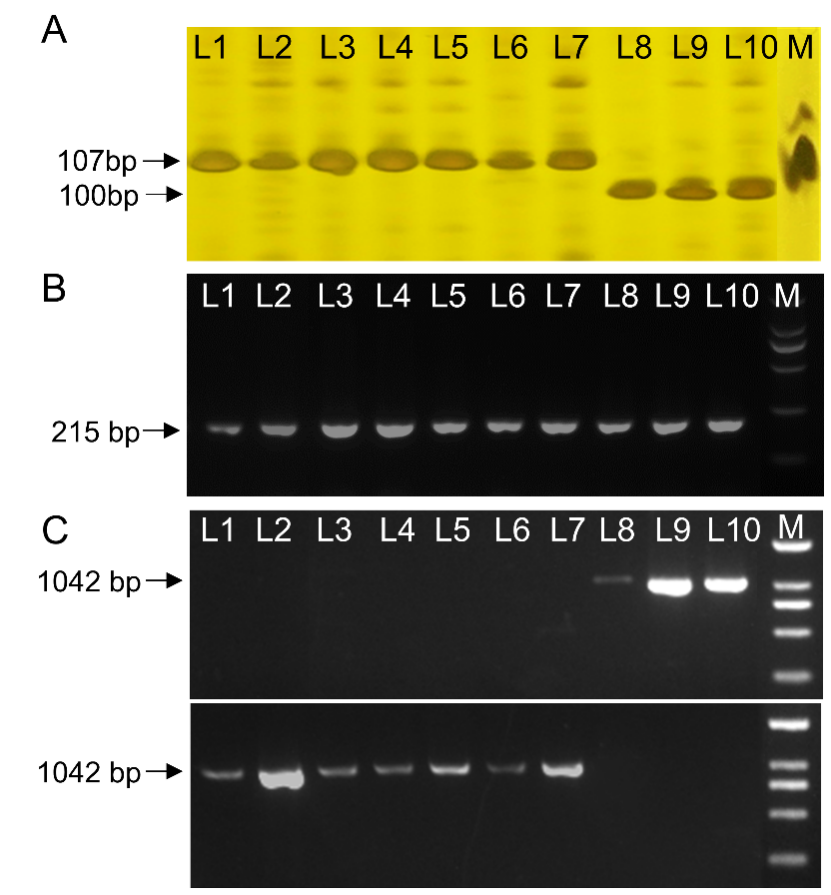


**Supplementary Figure 5.** Molecular marker genotyping evidence for seven monogenic lines (L1-L7) of *Pigm* and three pyramiding lines (L8-L10) of *Pigm*, *Pi-ta*, and *badh2*. The genotyping results of *badh2* **(A)**, *Pigm* **(B),** and *Pi-ta* **(C)** were identified by the molecular marker InDel-E2, M143104, and YL155/YL87 (top of C) and YL183/YL87 (bottom of C). M, marker DL2000.


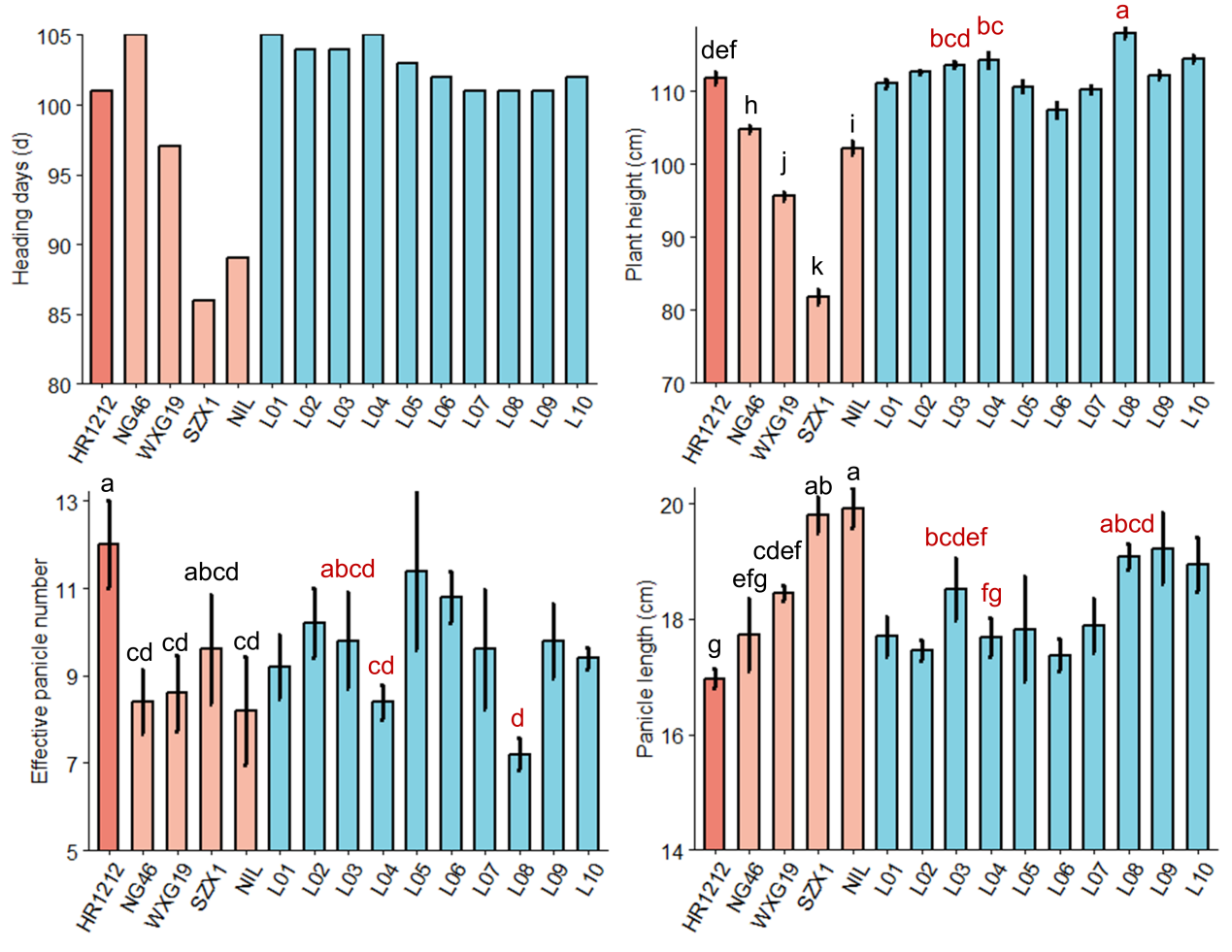


**Supplementary Figure 6.** Comparison of main agronomic traits among the introgressed lines and the parents. The red letters correspond to the top three resistant introgressed lines for rice blast. Data was presented as mean ± standard errors, *n*=5. Multiple comparison was conducted using the least significant difference (LSD) test. Different letters above the bars indicate significant differences among them (*P*<0.05).

**
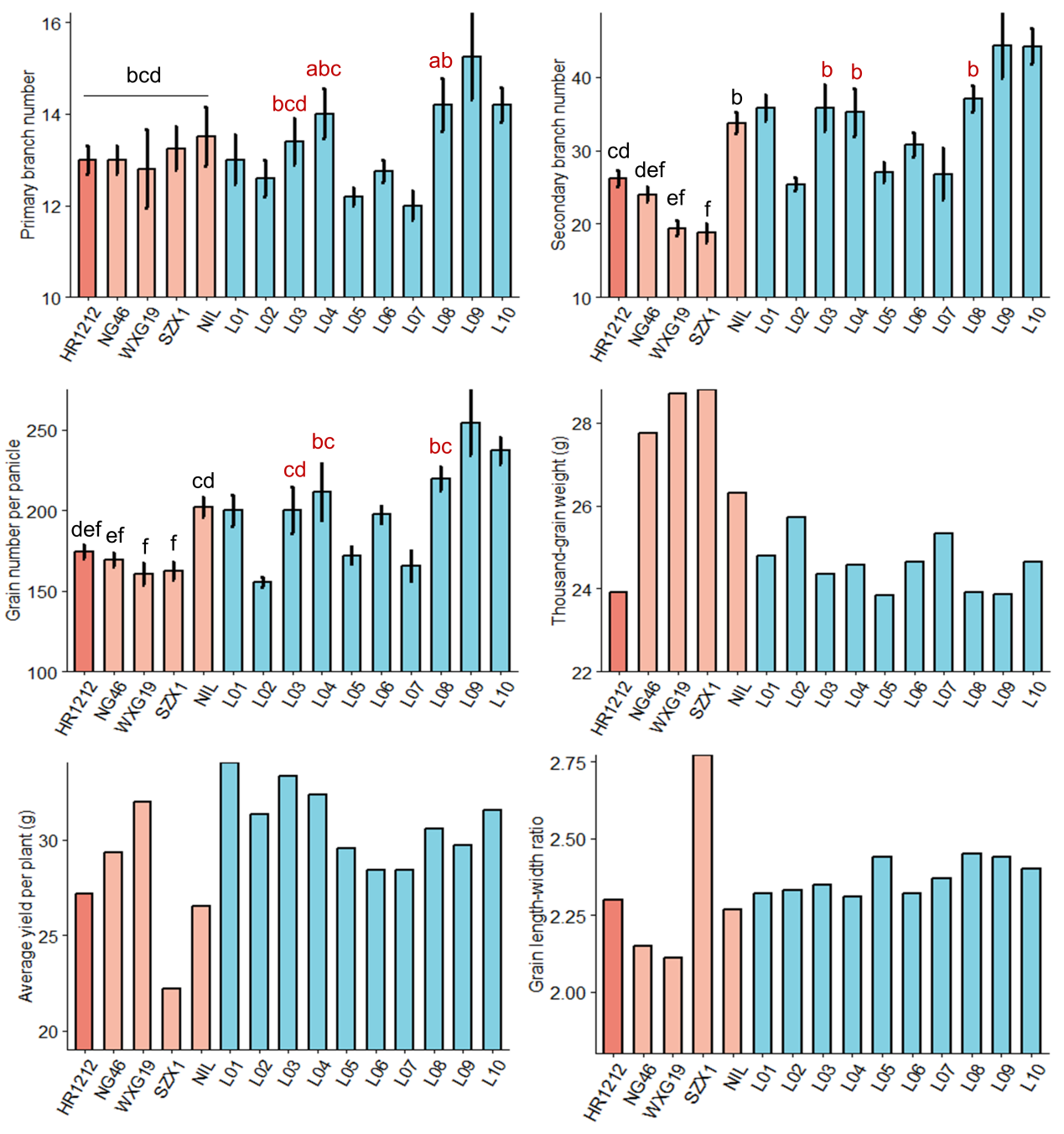
**

**Supplementary Figure 7.** Comparison of yield-related traits among the introgressed lines and the parents. The red letters correspond to the top three resistant introgressed lines for rice blast. Data was presented as mean ± standard errors, *n*=5.Multiple comparison was conducted using the least significant difference (LSD) test. Different letters above the bars indicate significant differences among them (*P*<0.05).


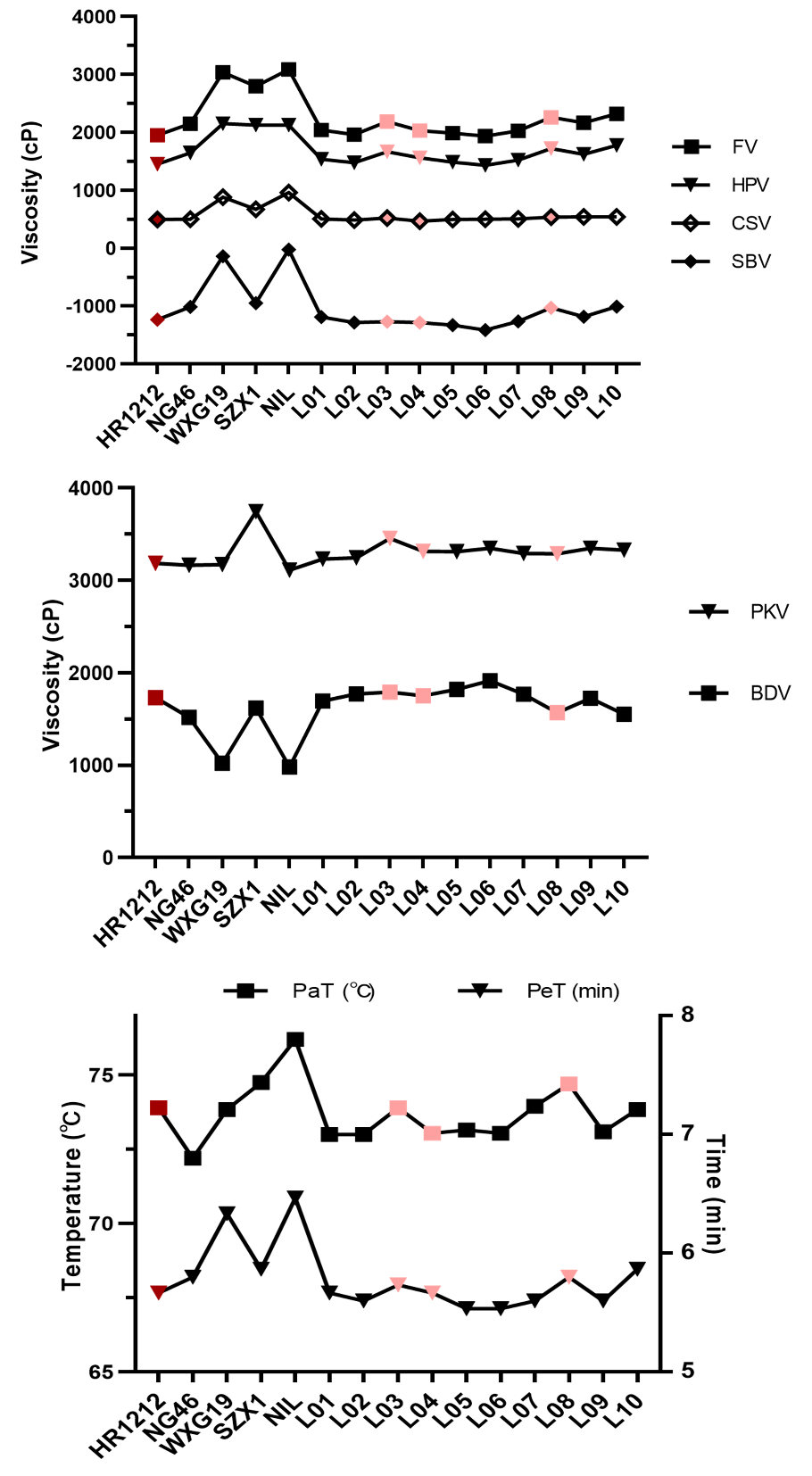


**Supplementary Figure 8.** Comparison of RVA profile properties among the introgressed lines and the parents. HPV, hot paste viscosity; FV, final viscosity; SBV, setback viscosity; CSV, consistence viscosity; PKV, peak viscosity; BDV, breakdown viscosity; PaT, pasting temperature; PeT, peak time. The dark red point and pink points correspond to HR1212 and the three most resistant introgressed lines for rice blast, respectively.


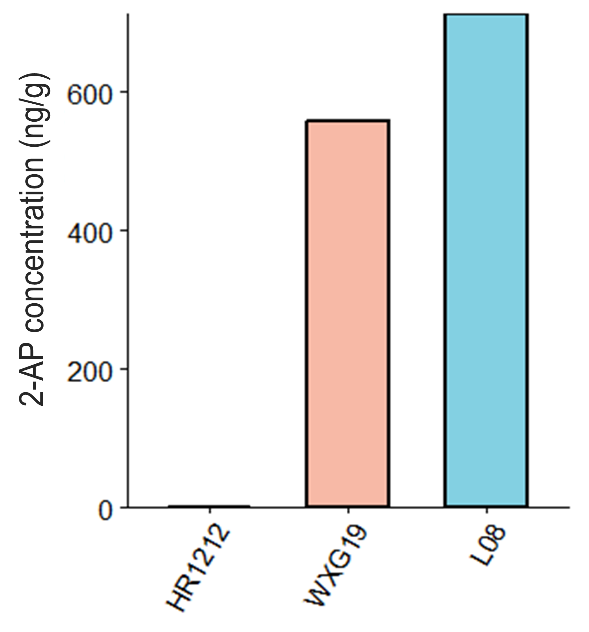


**Supplementary Figure 9.** The 2-AP concentration was measured in the milled rice

## Supplementary Tables

**Supplementary Table 1.** Molecular markers for genotyping of *Pigm*, *badh2* and *Pi-ta* in this study.

| Gene/  Locus | Marker | Chr | Primer sequence | | AT (℃) | ES (bp) | Reference |
| --- | --- | --- | --- | --- | --- | --- | --- |
|  |  |  | Forward (5'-3') | Reverse (5'-3') |  |  |  |
| *Pigm* | M143104 | 6 | CCTTGTTCCTCCTGCTATC | ATCTCGCTGTTCAGTCTTG | 56 | 215 | Wang et al., 2023 |
| *badh2* | InDel-E2 | 8 | GGGAGGCGCTGAAGAGGA | GGGTAGTCACCACCCTACCTTG | 60 | 100 | Wang et al., 2008 |
| *Pi-ta* | YL155/YL87 | 12 | AGCAGGTTATAAGCTAGGCC | CTACCAACAAGTTCATCAAA | 55 | 1042 | Jia et al., 2002 |
|  | YL183/YL87 |  | AGCAGGTTATAAGCTAGCTAT | CTACCAACAAGTTCATCAAA | 55 | - | Jia et al., 2004 |

Chr, Chromosome; AT, Annealing temperature; ES, Expected size.

**Supplementary Table 2.** Summary of molecular marker detection in multiple generations of Population 1 (P1) and Population 2 (P2).

| Population | Backcross generation | Genotype^a^ | *Pi-ta* | | *badh2* | | Number of plants carrying favorable alleles of *Pi-ta* and *badh2*^b^ |
| --- | --- | --- | --- | --- | --- | --- | --- |
|  |  |  | Count | Ratio (%) | Count | Ratio (%) |  |
| P1 | BC_2_F_1_ | FG | 0 | 0.00 | 0 | 0.00 | 16 |
|  |  | H | 24 | 40.68 | 20 | 34.48 |  |
|  |  | IG | 35 | 59.32 | 38 | 65.52 |  |
|  | BC_4_F_1_ | FG | 0 | 0.00 | 0 | 0.00 | 40 |
|  |  | H | 213 | 39.96 | 41 | 27.52 |  |
|  |  | IG | 320 | 60.04 | 108 | 72.48 |  |
| P2 | BC_1_F_1_ | FG | 0 | 0.00 | 0 | 0.00 | 26 |
|  |  | H | 39 | 54.93 | 40 | 57.14 |  |
|  |  | IG | 32 | 45.07 | 30 | 42.86 |  |
|  | BC_3_F_1_ | FG | 0 | 0.00 | 0 | 0.00 | 101 |
|  |  | H | 347 | 37.03 | 103 | 30.47 |  |
|  |  | IG | 590 | 62.97 | 235 | 69.53 |  |
|  | BC_4_F_1_ | FG | 0 | 0.00 | 0 | 0.00 | 45 |
|  |  | H | 213 | 39.96 | 45 | 42.06 |  |
|  |  | IG | 320 | 60.04 | 62 | 57.94 |  |
|  | BC_5_F_1_ | FG | 0 | 0.00 | 0 | 0.00 | 35 |
|  |  | H | 49 | 40.16 | 46 | 44.66 |  |
|  |  | IG | 73 | 59.84 | 57 | 55.34 |  |

^a^ FG: Favorable homozygous genotype. H: Heterozygous genotype. IG: Inferior homozygous genotype.

^b^ The favorable alleles here are all heterozygotes.

**Supplementary Table 3.** Summary of *Pigm* genotyping results in generations of Population 3 (P3).

| Genotype^a^ | BC_1_F_1_ | | BC_2_F_1_ | | BC_4_F_1_ | | BC_6_F_1_ | |
| --- | --- | --- | --- | --- | --- | --- | --- | --- |
|  | Count | Ratio (%) | Count | Ratio (%) | Count | Ratio (%) | Count | Ratio (%) |
| FG | 0 | 0.00 | 0 | 0.00 | 0 | 0.00 | 0 | 0.00 |
| H | 9 | 37.50 | 62 | 46.97 | 921 | 47.62 | 24 | 42.11 |
| IG | 15 | 62.50 | 70 | 53.03 | 1013 | 52.38 | 47 | 82.46 |

^a^ FG: Favorable homozygous genotype. H: Heterozygous genotype. IG: Inferior homozygous genotype.

**Supplementary Table 4.** Statistics on the individuals carrying favorable alleles in the progeny of crosses.

| Number of favorable alleles^a^ | Favorable allele(s)^a^ | P1-BC_5_F_1_×  P3-BC_5_F_1_ | P3-BC_5_F_1_×  P2-BC_5_F_1_ | P3-BC_6_F_1_ |
| --- | --- | --- | --- | --- |
|  |  |  |  |  |
| Three loci |  | 5 | 1 | - |
|  | *Pigm*, *Pi-ta*, *badh2* | 5 | 1 | - |
| Two loci |  | 18 | 4 | - |
|  | *Pigm, Pi-ta* | 8 | 2 | - |
|  | *Pigm, badh2* | 7 | 0 | - |
|  | *Pi-ta, badh2* | 3 | 2 | - |
| One locus |  | 20 | 7 | 24 |
|  | *Pigm* | 11 | 6 | 24 |
|  | *Pi-ta* | 8 | 1 | - |
|  | *badh2* | 1 | 0 | - |

^a^ The favorable alleles here are all in the form of heterozygotes.

**Supplementary Table 5.** The pedigree of the 41 individuals selected and the favorable allele(s) they carry.

| **Source** | **Pedigree** | **Favorable allele(s)^a^** | **Number of individuals** |
| --- | --- | --- | --- |
| P3-BC_6_F_1_ | HR1212///////NIL | *Pigm* | 20 |
| P1-BC_5_F_1_×P3-BC_5_F_1_ | HR1212//////(NG46/WXG19)/HR1212//////NIL | *Pigm* | 3 |
|  |  | *Pigm, Pi-ta* | 4 |
|  |  | *Pigm, badh2* | 4 |
|  |  | *Pigm, Pi-ta, badh2* | 3 |
| P3-BC_5_F_1_×P2-BC_5_F_1_ | HR1212//////NIL/HR1212/////SZX1 | *Pigm* | 4 |
|  |  | *Pigm, Pi-ta* | 2 |
|  |  | *Pigm, Pi-ta, badh2* | 1 |

^a^ The favorable alleles here are all in the form of heterozygotes.

**Supplementary Table 6.** Basic information of re-sequencing results of 41 introgressed individuals and their recurrent parent.

| Sample | Source | Raw reads | Raw bases (bp) | Clean reads | Clean | Mapping | GC | Average |
| --- | --- | --- | --- | --- | --- | --- | --- | --- |
|  |  |  |  |  | Q30 (%) | rate (%) | Content (%) | depth |
| Huruan1212 | - | 97,867,056 | 14,680,058,400 | 97,241,141 | 93 | 99.38 | 42.5 | 38.5 |
| C01 | P3-BC6F1 | 47,185,902 | 7,077,885,300 | 45,819,812 | 94.18 | 82.78 | 46.34 | 13.87 |
| C02 | P3-BC6F1 | 47,185,902 | 7,077,885,300 | 45,628,712 | 93.97 | 77.44 | 46.12 | 12.89 |
| C03 | P3-BC6F1 | 48,933,528 | 7,340,029,200 | 47,554,230 | 94.8 | 82.5 | 45.34 | 14.38 |
| C04 | P3-BC6F1 | 47,185,902 | 7,077,885,300 | 45,790,288 | 94.99 | 86.04 | 44.75 | 14.48 |
| C05 | P3-BC6F1 | 47,185,902 | 7,077,885,300 | 45,925,444 | 95.01 | 82.91 | 45.61 | 13.97 |
| C06 | P3-BC6F1 | 48,933,528 | 7,340,029,200 | 47,626,736 | 95.57 | 86.5 | 44.25 | 15.16 |
| C07 | P3-BC6F1 | 48,933,528 | 7,340,029,200 | 47,798,408 | 95.23 | 82.19 | 46.44 | 14.41 |
| C08 | P3-BC6F1 | 47,185,902 | 7,077,885,300 | 45,345,886 | 94.72 | 83.72 | 45.06 | 13.92 |
| C09 | P3-BC6F1 | 48,933,528 | 7,340,029,200 | 47,629,132 | 94.57 | 75.63 | 47.83 | 13.12 |
| C10 | P3-BC6F1 | 47,185,902 | 7,077,885,300 | 45,714,744 | 94.76 | 72.29 | 47.13 | 12.05 |
| C11 | P3-BC6F1 | 48,933,528 | 7,340,029,200 | 48,032,562 | 94.45 | 83.25 | 45.89 | 14.74 |
| C12 | P3-BC6F1 | 47,185,902 | 7,077,885,300 | 45,964,044 | 95.15 | 79.09 | 45.93 | 13.3 |
| C13 | P3-BC6F1 | 48,933,528 | 7,340,029,200 | 47,738,940 | 94.44 | 85.83 | 44.99 | 15.13 |
| C14 | P3-BC6F1 | 48,933,528 | 7,340,029,200 | 47,635,576 | 94.52 | 81.54 | 45.79 | 14.26 |
| C15 | P3-BC6F1 | 47,185,902 | 7,077,885,300 | 45,944,416 | 95.22 | 79.81 | 46.3 | 13.36 |
| C16 | P3-BC6F1 | 50,681,154 | 7,602,173,100 | 49,367,170 | 95.16 | 80.32 | 46.49 | 14.54 |
| C17 | P3-BC6F1 | 47,185,902 | 7,077,885,300 | 45,938,730 | 93.68 | 85.38 | 45.38 | 14.45 |
| C18 | P3-BC6F1 | 48,933,528 | 7,340,029,200 | 47,979,334 | 94.14 | 84.76 | 45.01 | 15.01 |
| C19 | P3-BC6F1 | 47,185,902 | 7,077,885,300 | 45,858,972 | 93.99 | 76.85 | 47.28 | 12.92 |
| C20 | P3-BC6F1 | 50,681,154 | 7,602,173,100 | 49,152,678 | 94.19 | 83.83 | 45.66 | 15.12 |
| C21 | P1-BC5F1×P3-BC5F1 | 48,933,528 | 7,340,029,200 | 47,773,994 | 93.7 | 80.04 | 46.19 | 14.08 |
| C22 | P1-BC5F1×P3-BC5F1 | 48,933,528 | 7,340,029,200 | 47,578,914 | 94.42 | 77.9 | 46.52 | 13.57 |
| C23 | P1-BC5F1×P3-BC5F1 | 48,933,528 | 7,340,029,200 | 47,483,722 | 94.16 | 77.68 | 46.84 | 13.39 |
| C24 | P3-BC5F1×P2-BC5F1 | 47,185,902 | 7,077,885,300 | 45,606,034 | 93.82 | 71.79 | 48.59 | 11.88 |
| C25 | P3-BC5F1×P2-BC5F1 | 47,185,902 | 7,077,885,300 | 45,554,688 | 94.25 | 76.45 | 47.65 | 12.7 |
| C26 | P3-BC5F1×P2-BC5F1 | 48,933,528 | 7,340,029,200 | 46,489,808 | 94.1 | 80.08 | 46.92 | 13.56 |
| C27 | P3-BC5F1×P2-BC5F1 | 47,185,902 | 7,077,885,300 | 45,201,302 | 94.3 | 83.01 | 45.99 | 13.7 |
| C28 | P1-BC5F1×P3-BC5F1 | 47,185,902 | 7,077,885,300 | 45,624,954 | 94.1 | 75.5 | 47.57 | 12.41 |
| C29 | P1-BC5F1×P3-BC5F1 | 50,681,154 | 7,602,173,100 | 49,553,994 | 93.52 | 81.17 | 45.63 | 14.8 |
| C30 | P1-BC5F1×P3-BC5F1 | 47,185,902 | 7,077,885,300 | 44,852,744 | 93.46 | 80.41 | 47.02 | 13.02 |
| C31 | P1-BC5F1×P3-BC5F1 | 50,681,154 | 7,602,173,100 | 47,410,994 | 93.43 | 84.04 | 45.49 | 14.54 |
| C32 | P3-BC5F1×P2-BC5F1 | 50,681,154 | 7,602,173,100 | 48,042,812 | 93.55 | 83.14 | 45.49 | 14.53 |
| C33 | P3-BC5F1×P2-BC5F1 | 48,933,528 | 7,340,029,200 | 46,641,634 | 93.05 | 82.06 | 45.59 | 14.01 |
| C34 | P1-BC5F1×P3-BC5F1 | 50,681,154 | 7,602,173,100 | 48,492,452 | 93.22 | 79.69 | 47.16 | 14.02 |
| C35 | P1-BC5F1×P3-BC5F1 | 47,185,902 | 7,077,885,300 | 45,333,202 | 93.24 | 81.27 | 45.96 | 13.48 |
| C36 | P1-BC5F1×P3-BC5F1 | 47,185,902 | 7,077,885,300 | 45,134,566 | 93.2 | 85 | 45.34 | 14.05 |
| C37 | P1-BC5F1×P3-BC5F1 | 48,933,528 | 7,340,029,200 | 47,346,376 | 94.35 | 76.68 | 47.2 | 13.09 |
| C38 | P1-BC5F1×P3-BC5F1 | 50,681,154 | 7,602,173,100 | 48,653,880 | 94.93 | 78.57 | 46.43 | 13.81 |
| C39 | P1-BC5F1×P3-BC5F1 | 52,428,780 | 7,864,317,000 | 46,530,690 | 94.98 | 82.85 | 47.29 | 13.94 |
| C40 | P1-BC5F1×P3-BC5F1 | 47,185,902 | 7,077,885,300 | 45,263,830 | 94.43 | 84.73 | 45.48 | 14.04 |
| C41 | P3-BC5F1×P2-BC5F1 | 48,933,528 | 7,340,029,200 | 47,590,474 | 94.45 | 82.42 | 45.84 | 14.37 |
